# Supplementary material for: Contributions of anterior cingulate cortex and basolateral amygdala to decision confidence and learning under uncertainty
Source: Nat Commun. 2019 Oct 17;10:4704. doi: 10.1038/s41467-019-12725-1 (PMC6797780; doi:10.1038/s41467-019-12725-1)
Supplement: Supplementary file 1 — Supplementary Information [file 41467_2019_12725_MOESM1_ESM.pdf]

Supplementary Information

Supplementary Notes 1: Accuracy increases for easier, more discriminable stimuli. We analyzed the effect of contrast and SNR on accuracy. First, we separated the trials by contrast and computed accuracy as a function of SNR. We observed that accuracy increases as SNR increases for each of the contrast levels (Supplementary Fig. 1A; GLM contrast=40: $p = 1.14 \times 10^{-27}$; $\beta_{SNR} = 0.108,\ p = 1 \times 10^{-3}$; GLM contrast=60: $p = 6.07 \times 10^{-32}$; $\beta_{SNR} = 0.157,\ p = 2.08 \times 10^{-8}$; GLM contrast=80: $p = 1.19 \times 10^{-11}$; $\beta_{SNR} = 0.087,\ p = 2 \times 10^{-3}$). Observing the effect for each contrast, we also calculated accuracy as a function of SNR across all contrast values. Similarly, we found that accuracy increases with SNR on accuracy (Supplementary Fig. 1B; GLM: $p = 5.01 \times 10^{-70}$; $\beta_{SNR} = 0.118,\ p = 1.23 \times 10^{-11}$). These results illustrate that for easier stimuli (higher SNR), performance is better compared to the more difficult stimuli (lower SNR).

Supplementary Notes 2: Trial re-initiation is only affected by SNR. In order to ensure that our drug manipulations did not change the tendency of the animals to re-initiate a new trial, we used a GLM to examine whether the number of re-initiations was affected by either vehicle or CNO administration, or by either the BLA and ACC as the targeted brain region, or SNR of the stimuli. Importantly, we found that only SNR significantly affected the number of trial re-initiations (GLM: $p = 1.14 \times 10^{-16}$; $\beta_{\text{ratio}} = -2.26, p = 0.00026$), indicating that re-initiation mainly depended on the strength of the visual information and thus perceptual uncertainty.

Supplementary Notes 3: Normalized waiting and reaction times. We repeated our main analyses using z-scored waiting time and reaction time to confirm that the observations were not due to session-based variability of behavior or driven by individual differences. First, we found that z-scored waiting times were longer on trials with greater SNR (GLM: $p = 5.68 \times 10^{-242}$; $\beta_{SNR} = 0.23, p = 1.84 \times 10^{-24}$). In contrast, z-scored reaction time decreased with increased SNR (GLM: $p = 5.89 \times 10^{-224}$; $\beta_{SNR} = -0.46, p = 2.83 \times 10^{-81}$). Additionally, z-scored waiting times were negatively correlated with z-scored reaction times on a trial-by-trial basis (GLM: $p = 1.98 \times 10^{-161}$; $\beta_{RT} = -0.21, p = 1.98 \times 10^{-161}$). To examine the relationship between accuracy and time wagering, we also computed waiting time for correct and

incorrect responses. We found that rats waited significantly longer following correct relative to incorrect responses, or trial type (diff(mean)=0.58; GLM: $p = 1.83 \times 10^{-78}$; $\beta_{\text{trial type}} = 0.56, p = 6.24 \times 10^{-41}$). In addition, waiting times on both correct and incorrect responses increased with greater SNR (GLM Correct: $p = 7.81 \times 10^{-102}$; $\beta_{SNR} = 0.45,\ p = 6.95 \times 10^{-58}$; GLM Incorrect: $p = 3.09 \times 10^{-48}$; $\beta_{SNR} = 0.23,\ p = 5.39 \times 10^{-28}$). These results demonstrate that not only is z-scored waiting time sensitive to the strength of the visual information, but it also reflects rats' accuracy in discrimination. Finally, we also found that z-scored reaction times decreased with greater SNR and tended to be faster for correct responses relative to incorrect responses/discrimination (diff(mean)=-0.08; GLM: $p = 0.06$; $\beta_{SNR} = -0.094, p = 0.03$). Overall, the results based on z-scored waiting time and reaction time yielded an identical pattern to our previous results. Importantly, this also holds true for the results of inhibition of ACC and BLA.

Supplementary Notes 4: Vehicle administration has no impact on its own. To show that vehicle administration alone does not produce the observed behavioral changes, we compared the effect of vehicle administration and no-injection on waiting time and reaction time. Similar to vehicle administration, we found that in the sessions in which no injection was administered, waiting times were longer on trials with a larger SNR (GLM: no-injection: $p = 1.26 \times 10^{-185}$; $r = 1.75, p = 2.46 \times 10^{-15}$; Supplementary Fig. 8). Furthermore, waiting times were negatively correlated with reaction times on a trial-by-trial basis (GLM: ACC inhibition: slope= $-0.62, p = 2.83 \times 10^{-6}$; BLA inhibition: slope= $-1.43, p = 2.88 \times 10^{-47}$; no-injection: slope= $-2.53, p = 3.02 \times 10^{-162}$).

Supplementary Notes 5: Absence of non-specific effects of virus exposure. In the main body of the manuscript we presented data on behavior following vehicle and CNO administration in rats with DREADDs expressed in the BLA and ACC. Here, we include data demonstrating that these impairments are not due to non-specific effects of surgery/virus exposure. Therefore, in this section we compare null (EGFP) and active (DREADDs) virus following vehicle administration on the major measures of the task such as waiting time, reaction time, performance, d', and meta-d'/d'.

We first observed that the type of virus (null vs. active) had no significant main effect or interaction with trial type (correct vs. incorrect) and/or SNR values on waiting time (GLM: $p = 6.12 \times 10^{-57}$; $\beta_{\text{virus type}} = 1.61, p = 0.3$; $\beta_{\text{virus type} \times \text{trial type}} = -0.16, p = 0.94$; $\beta_{\text{virus type} \times \text{ratio}} = -0.008, p = 0.98$; $\beta_{\text{virus type} \times \text{trial type} \times \text{ratio}} = -0.35, p = 0.62$; Supplementary Fig. 9A). Similarly, we did not find a significant effect of virus type in either main effect or interactions with trial type and SNR on reaction time (GLM: $p = 9.02 \times 10^{-77}$; $\beta_{\text{virus type}} = 0.09, p = 0.23$; $\beta_{\text{virus type} \times \text{trial type}} = -0.13, p = 0.25$; $\beta_{\text{virus type} \times \text{ratio}} = -0.03, p = 0.15$; $\beta_{\text{virus type} \times \text{trial type} \times \text{ratio}} = 0.03, p = 0.44$; Supplementary Fig. 9B).

Next, we analyzed the effect of virus type on performance measures of probability of correct response, d', and meta-d'/d'. We observed no significant effect of virus type or interaction with SNR on probability of correct response (GLM: $p = 2.16 \times 10^{-23}$; $\beta_{\text{virus type}} = -0.03, p = 0.15$; $\beta_{\text{virus type} \times \text{ratio}} = 0.01, p = 0.16$; Supplementary Fig. 9C**).** Furthermore, we observed that the type of virus had no significant effect or interaction with SNR values on d' (GLM: $p = 2.13 \times 10^{-45}$; $\beta_{\text{virus type}} = -0.35, p = 0.6$; $\beta_{\text{virus type} \times \text{ratio}} = 0.15, p = 0.48$; Supplementary Fig. 9D). Finally, we observed no significant effect of virus type or interaction with ratio on meta-d'/d' (GLM: $p = 0.005$; $\beta_{\text{virus type}} = -1.01, p = 0.27$; $\beta_{\text{virus type} \times \text{ratio}} = 0.12, p = 0.68$; Supplementary Fig. 9E). These results show that the observed impairments through CNO administration was specific to the active (DREADDs) virus we used in the experiment.

Supplementary Notes 6: Relationship between waiting time and reaction time in different experimental conditions. The confidence intervals (95%) for the slopes of linear regressors indicated that the negative correlations between waiting time and reaction time in the two control conditions (vehicle and no-injection prior to reversal) were not significantly different, but were significantly different following CNO administration. More specifically, the confidence intervals for the slopes are as follows: vehicle administration, $[-2.7061, -2.3402]$; ACC inhibition, $[-0.8850, -0.3630]$; BLA inhibition, $[-1.6288, -1.2418]$; no-injection, $[-2.7086, -2.3478]$ (Supplementary Fig. 10). Thus, the correlation between these measures was still negative but weaker in both ACC and BLA inhibition conditions. In addition, correlation between waiting time and reaction time was weaker after ACC than BLA inhibition.

Supplementary Notes 7: Two vehicle groups with ACC and BLA as targeted brain region were similar in behavior. We calculated session-based waiting times following ACC inhibition and BLA inhibition and compared their medians with their corresponding vehicle groups (Supplementary Fig. 12). We found no significant differences between targeted brain regions following vehicle on waiting time (GLM: $p = 10^{-16}$; $\beta_{\text{region}} = 0.67, p = 0.83$). Furthermore, we separately analyzed trials in which rats responded correctly. As above, we found no significant differences between targeted brain regions following vehicle on waiting time (GLM: $p = 10^{-16}$; $\beta_{\text{region}} = -0.77, p = 0.89$). Next, we separately analyzed trials in which rats responded incorrectly to the trial. We similarly found no significant effect of targeted brain region following vehicle on waiting time (GLM: $p = 1.45 \times 10^{-228}$; $\beta_{\text{region}} = 2.12, p = 0.55$).

We also separately analyzed stimuli based on the contrast and found no significant differences in waiting time following vehicle administration in either ACC or BLA (Supplementary Fig. 13; GLM contrast = 40: $p = 1.27 \times 10^{-136}$; $\beta_{\text{region}} = -1.461, p = 0.26$; GLM contrast = 60: $p = 1.3 \times 10^{-260}$; $\beta_{\text{region}} = -0.7, p = 0.63$; GLM contrast = 80: $p = 10^{-16}$; $\beta_{\text{region}} = -2.66, p = 0.11$). Together, these results show that averaging the two vehicle groups is not masking a general effect of ACC inhibition observed in Figure 4. Furthermore, the vehicle waiting times were not significantly different between targeted regions.

Supplementary Notes 8: Effect of motivation on performance and waiting time. We performed additional analyses to examine whether changes in motivational state could explain the observed pattern of waiting time on correct and incorrect trials. First, we examined the effect of trial number on accuracy for high SNR (SNR = 4) and found no significant effect (GLM: $p = 4.73 \times 10^{-11}$; $\beta_{\text{trial}} = 0.001, p = 0.19$ ($> p^* = 0.05/3$)). Similarly, we did not find a significant effect of trial number on accuracy considering all SNR values together (GLM: $p = 7.05 \times 10^{-157}$; $\beta_{\text{trial}} = -0.0002, p = 0.9$). These analyses thus do not provide any evidence for differential distribution of errors throughout the session. Second, we found that z-scored waiting time did not significantly increase for high SNR as a function of trial number (GLM: $p = 3.11 \times 10^{-192}$; $\beta_{\text{trial}} = -0.0003, p = 0.8$). In addition, z-scored waiting time did not significantly increase as a function of trial number within a session (GLM: $p =$

1×10^{-170}; $\beta_{\text{trial}} = -0.0016, p = 0.49$). Therefore, we did not find any evidence that motivational state (which could decrease with time and satiation) influences accuracy. Together, our analyses illustrate that the observed increase in waiting time (confidence) on error trials with high SNR stimuli is likely not due to uneven distribution of errors throughout the session or due to a low motivational state.

Supplementary Notes 9: Quantification of viral spread and its influence. We quantified the spread of virus following hM4Di and eGFP infusions in both ACC and BLA. Tissue analysis was conducted by fluorescent pixel counts and also via an approximation of mm^2 spread. There was no significant difference in the spread between active vs. null virus ($p = 0.06$), no significant effect of region ($p = 0.57$), or virus by region interaction ($p = 0.45$). Additionally, we analyzed the effect of DREADDs spread on z-scored waiting time and accuracy following vehicle administration. We observed no significant main effect of spread on waiting time or accuracy (GLM waiting time: $p = 10^{-16}$; $\beta_{\text{spread}} = 0.4, p = 0.085$; GLM accuracy: $p = 1.65 \times 10^{-108}$; $\beta_{\text{spread}} = -0.26, p = 0.32$). Similarly, we found no significant main effect of DREADDs spread on waiting time or accuracy following CNO administration (GLM waiting time: $p = 10^{-16}$; $\beta_{\text{spread}} = -0.03, p = 0.89$; GLM accuracy: $p = 9.46 \times 10^{-120}$; $\beta_{\text{spread}} = -0.31, p = 0.24$). Together, these analyses do not provide any evidence that the spread of virus (null or active) influenced waiting time or accuracy. These results are in line with the fact that the spread of virus (either null or active) already covered the majority of the targeted regions for all rats and therefore no effect of spread size was expected.

Supplementary Notes 10: Effect of CNO depends on DREADDs. In order to study if CNO or its metabolic byproducts have an effect via a DREADDs-independent manner, we used the sessions in which null EGFP virus was infused. We first observed that there was no significant effect of drug administration type (vehicle vs. CNO) nor an interaction of administration with trial type (correct vs. incorrect) and/or SNR values on waiting time (GLM: $p = 1.25 \times 10^{-66}$; $\beta_{\text{administration}} = 0.12, p = 0.93$; $\beta_{\text{administration} \times \text{trial type}} = 0.30, p = 0.89$; $\beta_{\text{administration} \times \text{ratio}} = -0.21, p = 0.66$; $\beta_{\text{administration} \times \text{trial type} \times \text{ratio}} = 0.13, p = 0.84$; Supplementary Fig. 14A). In addition, we did not find a significant effect of drug administration

type or interactions of administration type with trial type and SNR on reaction time ($p = 3.63 \times 10^{-73}$; $\beta_{\text{administration}} = -0.01, p = 0.86$; $\beta_{\text{administration × trial type}} = 0.01, p = 0.91$; $\beta_{\text{administration × ratio}} = 0.003, p = 0.91$; $\beta_{\text{administration × trial type × ratio}} = -0.002, p = 0.94$; Supplementary Fig. 14B).

We also analyzed the effect of drug administration type on the performance measures of probability of correct response, d', and meta-d'/d'. We observed no significant effect of administration type or interaction with SNR on probability of correct response (GLM: $p = 1.64 \times 10^{-20}$; $\beta_{\text{administration}} = 0.003, p = 0.91$; $\beta_{\text{administration × ratio}} = -0.003, p = 0.60$; Supplementary Fig. 14C). Furthermore, we found no significant effect of administration type or interaction of administration type with SNR values on d' (GLM: $p = 1.7 \times 10^{-46}$; $\beta_{\text{administration}} = 0.40, p = 0.54$; $\beta_{\text{administration × ratio}} = -0.18, p = 0.38$; Supplementary Fig. 14D). Finally, we did not observe a significant effect of administration type or interaction with ratio on meta-d'/d' (GLM: $p = 0.43$; $\beta_{\text{administration}} = 0.04, p = 0.97$; $\beta_{\text{administration × ratio}} = -0.11, p = 0.74$; Supplementary Fig. 14E).

Together, these results do not provide any evidence that CNO (or its metabolic byproducts) act in a DREADDs-independent manner to account for the effects we report here.

Supplementary Figures

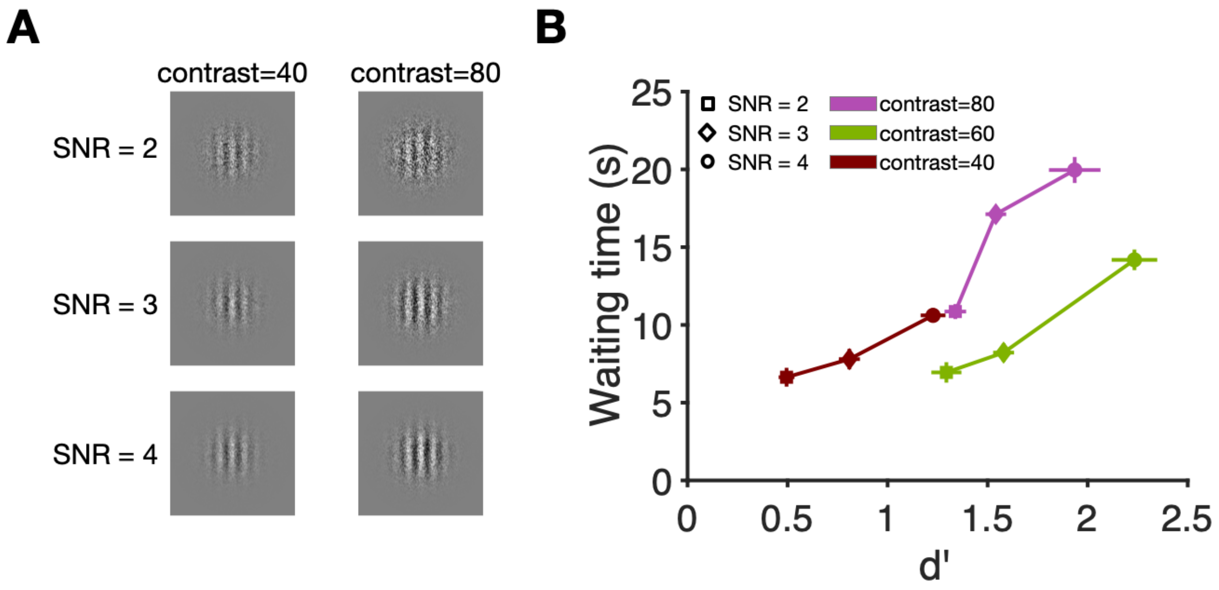

Supplementary Fig. 1. SNR, contrast of the visual stimuli, and waiting time and discrimination performance as a function of those manipulations. **(A)** Examples of visual stimuli with more (80) or less (40) contrast with different signal-to-noise (SNR) ratio, reflecting the strength of the visual signal (4, most discriminable; 3, moderately discriminable; 2, least discriminable). (**B**) Waiting time and d' increases with SNR for any value of contrast. Source data are provided as a Source Data file.

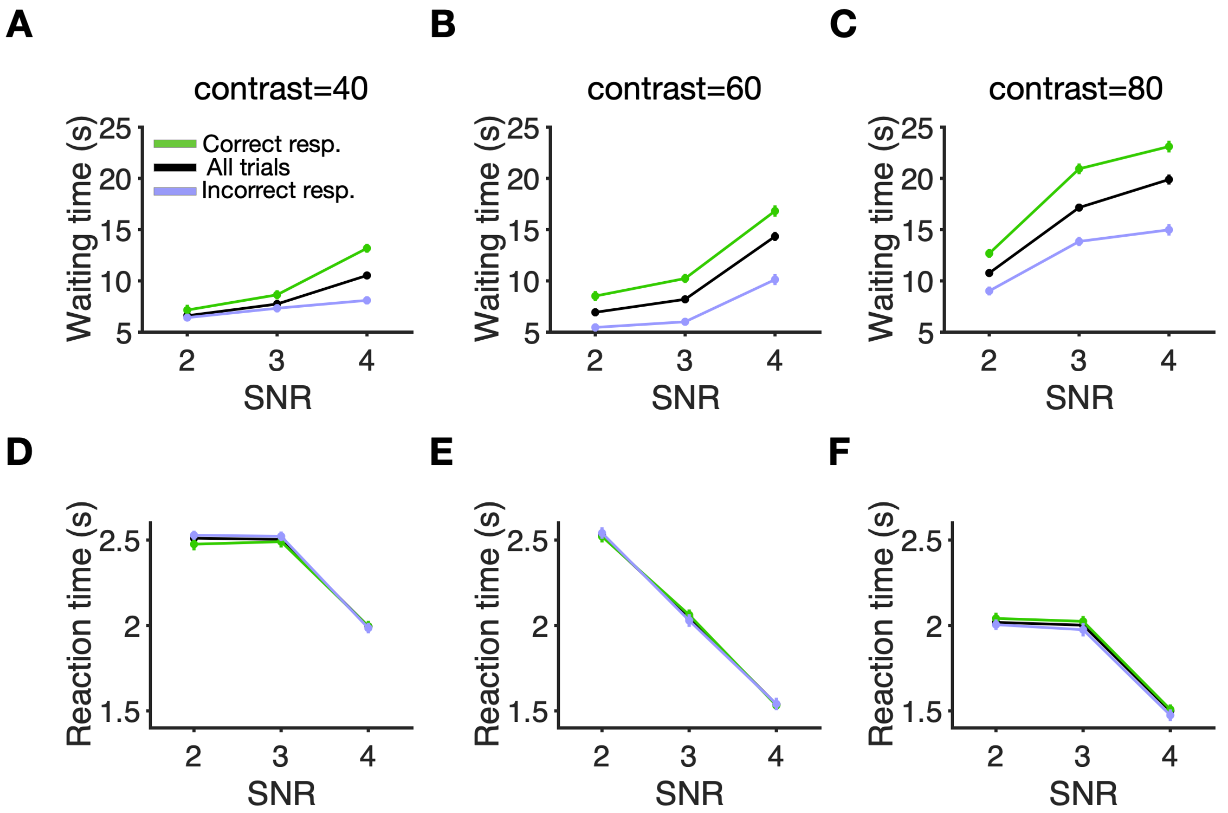

Supplementary Fig. 2. Waiting time increases with SNR for any value of contrast whereas reaction time dependence on SNR was strongly modulated by contrast. Plotted are the waiting time and reaction time as a function of SNR for different values of contrasts, and separately for correct and incorrect trials. **(A-C)** Plotted is the waiting time for all trials as a function of three contrast levels (40, weaker to 80, stronger) and different signal-to-noise (SNR) ratio, reflecting the strength of the visual signal (4, most discriminable; 3, moderately discriminable; 2, least discriminable) for correct responses (green), incorrect responses (blue), and all trials (black). (**D-E**) Same as in panel A-C but for reaction times. Error bars show the S.E.M. over sessions (typically smaller than the symbols). Source data are provided as a Source Data file.

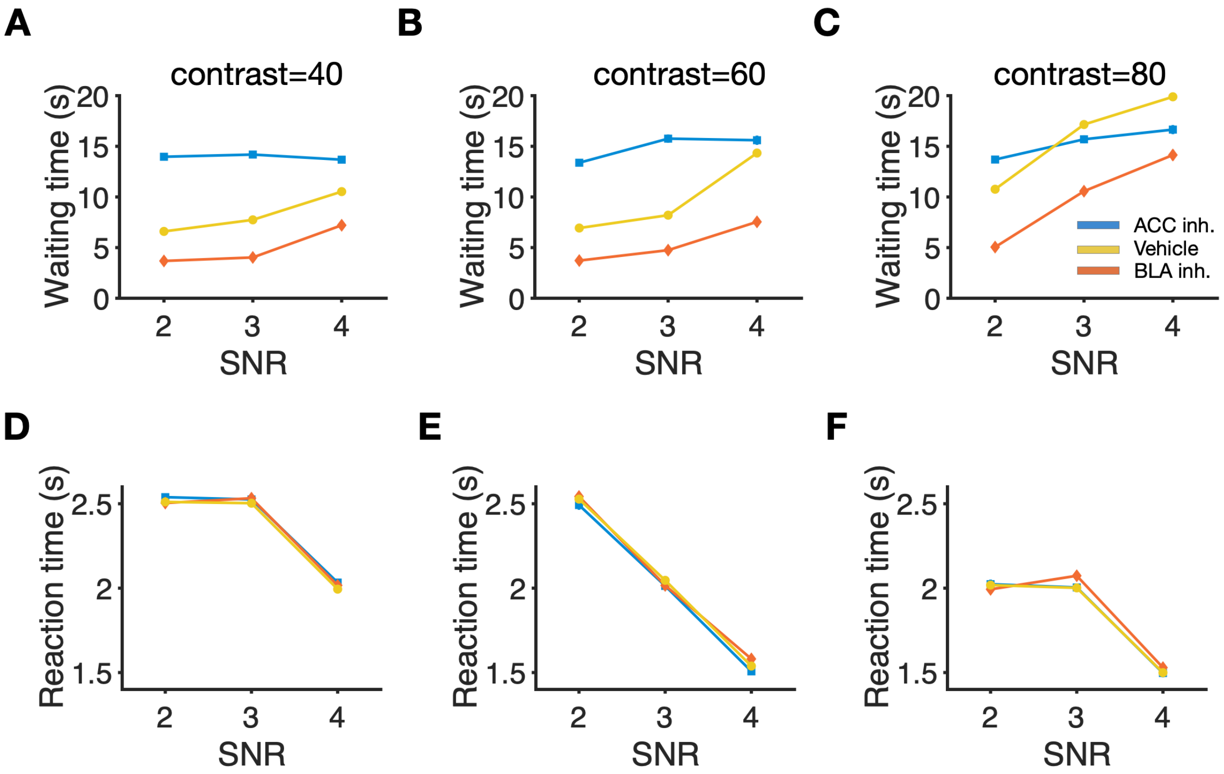

Supplementary Fig. 3. ACC and BLA inhibition influenced waiting time for all values of contrast whereas reaction time was not sensitive to these manipulations. Plotted are waiting time and reaction time as functions of SNR for different values of contrast after the inhibition of ACC and BLA. Conventions are the same as in Figure 3. Overall, ACC inhibition rendered waiting time insensitive to SNR for all contrast values such that at high contrast, waiting time in ACC-inhibited rats fell below the control condition. In contrast, BLA inhibition, reduced waiting time for all contrast values without reducing the sensitivity to SNR. Source data are provided as a Source Data file.

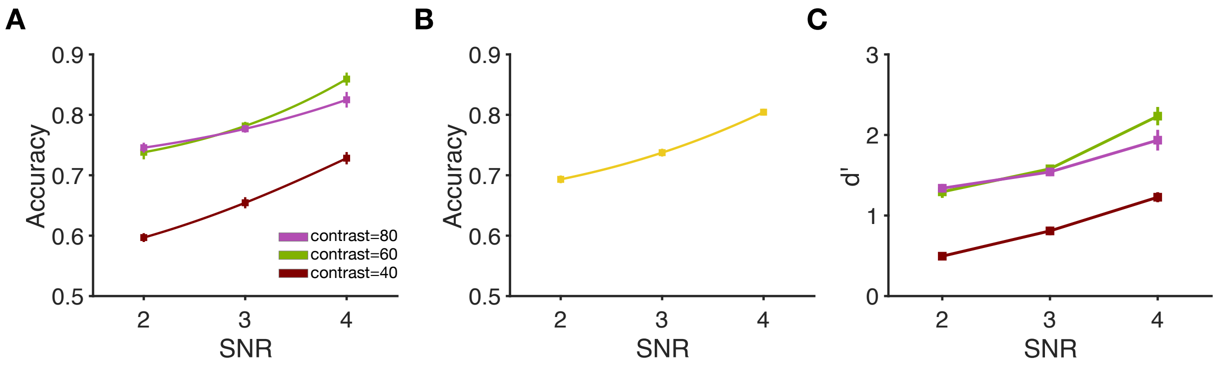

Supplementary Fig. 4. Accuracy increases for easier stimuli. (**A**) Accuracy is plotted as a function of SNR for different values of stimulus contrast. The squares and error bars show the mean and S.E.M. The solid lines show the fitted psychometric curve over the observed data. Different values of contrast are indicated in the legend and correspond to least discriminable (contrast = 40), moderately discriminable (contrast = 60), and most discriminable (contrast = 80). (**B**) Plots shows the average accuracy as a function of SNR across all contrast levels. (**C**) Plot shows d' as a function of SNR for different values of contrast. Source data are provided as a Source Data file.

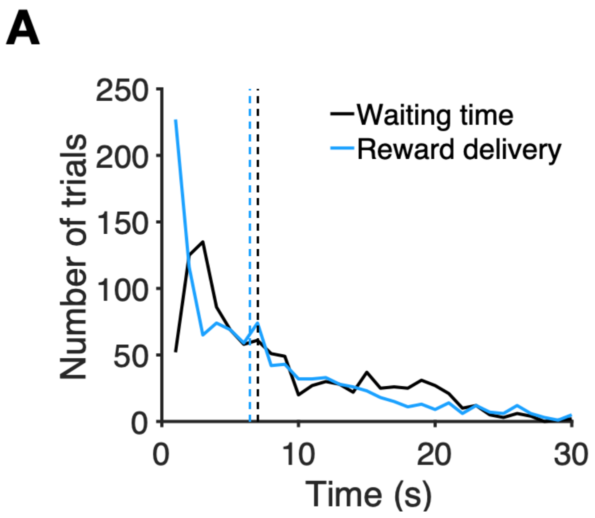

Supplementary Fig. 5. Rats' waiting time tracked distributions of the statistics of reward delivery time. Plotted are the distributions of reward delivery time (blue) and waiting time (black) in an example individual rat **(A)** and across all rats **(B)**. The dashed lines show the median of the reward delivery time distributions and of waiting time, respectively. The waiting time distribution generally followed that of the reward delivery time. Source data are provided as a Source Data file.

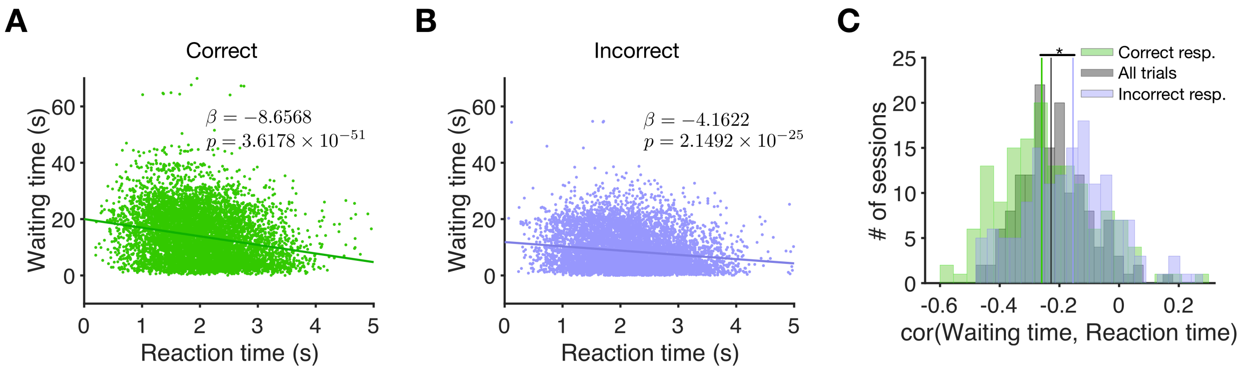

Supplementary Fig. 6. Waiting time before re-initiation of a new trial is negatively correlated with reaction time to make a choice. Waiting time is plotted as a function of the reaction time for all rats in correct trials **(A)** and incorrect trials **(B)**. Each data point is a trial in a session following vehicle administration. The correlation between waiting time and reaction time is significantly stronger in correct trials than in incorrect trials **(C)**. Plotted in (C) are distribution of the session-based correlation between waiting time and reaction time for all trials (black), correct trials (green), and incorrect trials (blue). Each solid line shows the median of each distribution. (*) indicates $p < 0.05$. Source data are provided as a Source Data file.

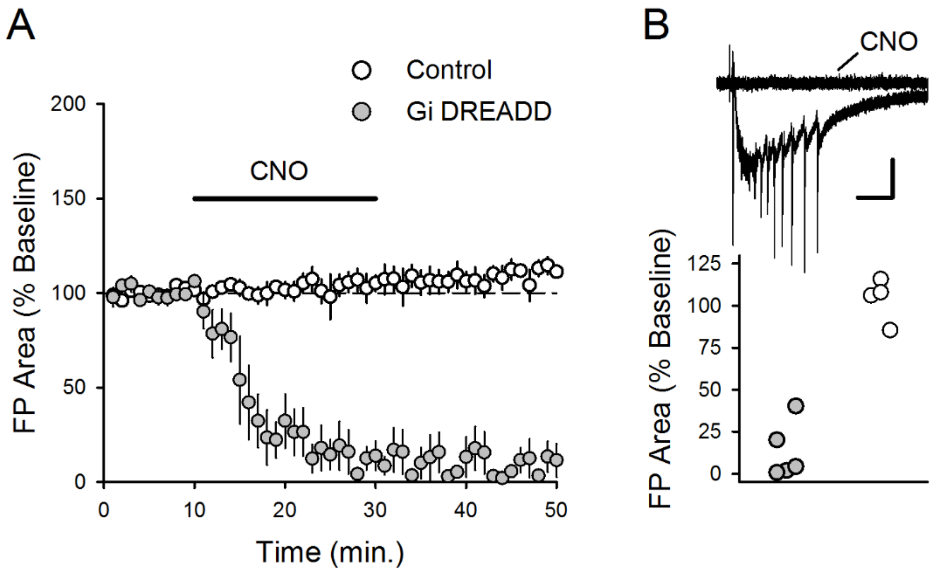

Supplementary Fig. 7. Electrophysiological effect of bath application of CNO in slice. (**A**) Inhibition of field potentials (FP) elicited by layer I stimulation followed by bath application of CNO (10 *μM*, indicated by the bar) in a control and DREADD preparation. Plot shows the FP area (normalized to baseline) over time. CNO was applied at t = 10 *min* (**B**) Top: Traces show superimposed responses elicited before and after CNO application in transfected slices. Calibration bars: 0.5 *mV*, 500 *msec*. Bottom: Points show FP area (normalized to baseline) at end of CNO application (averaged over last 5 *min*) from individual slices. Application of CNO strongly suppressed FPs in transfected slices (filled circles; FP area was reduced to 13.4 ± 8% (S.E.M) of baseline; paired t-test for comparison to baseline, $t(4) = 11.333$, $p = 3.46 \times 10^{-3}$, $n = 5$ slices from 3 rats) but had no effect on responses in non-transfected slices (open circles; FP area was 103.7 ± 7% of baseline; paired t-test for comparison to baseline, $t(3) = 0.578$, $p = 0.604$, $n = 4$ slices from 3 rats). Source data are provided as a Source Data file.

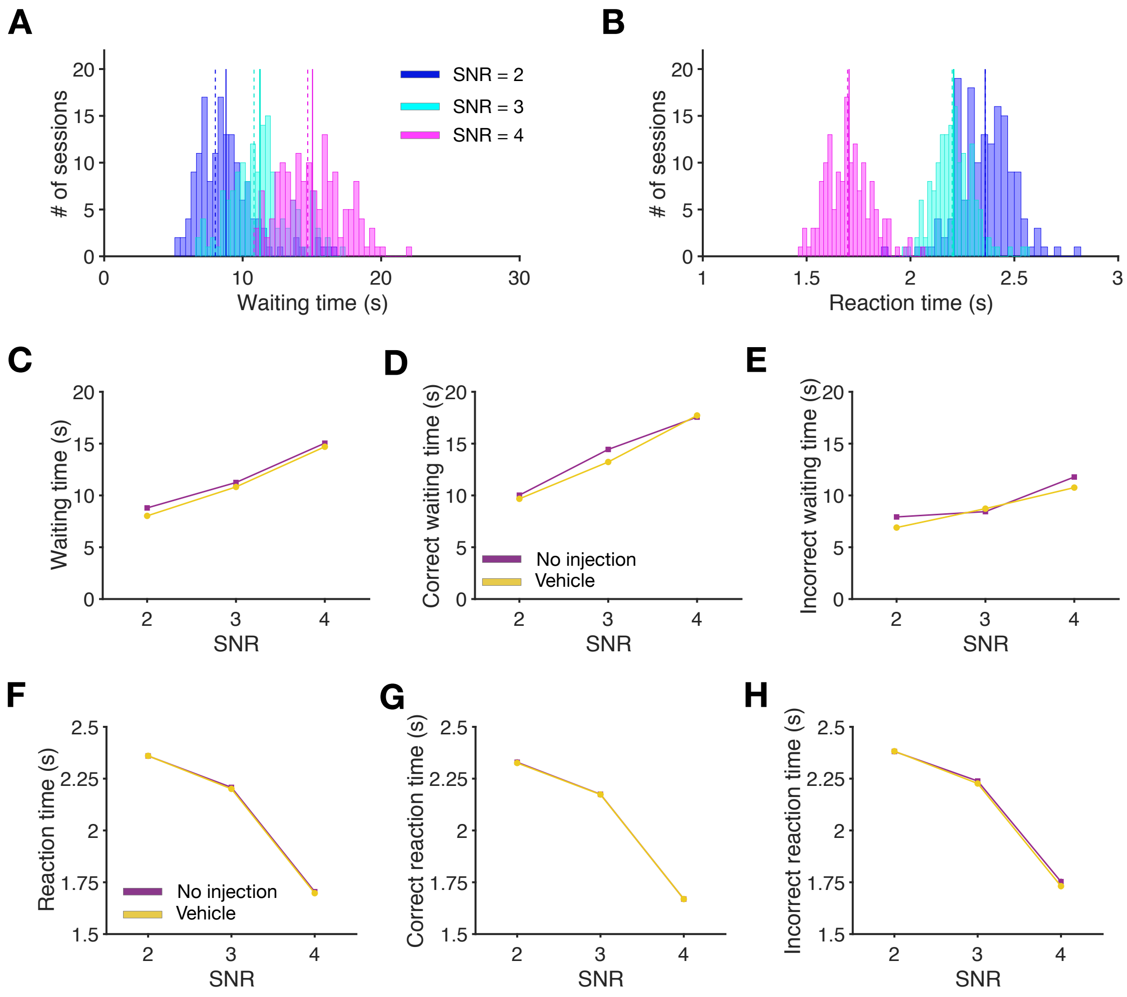

Supplementary Fig. 8. Behavior is similar following vehicle administration and no-injection control. **(A)** Waiting time in sessions with no injection increases similarly to sessions following vehicle administration. Plotted are the distributions of the waiting time for each SNR:2 (blue), 3 (cyan), and 4 (magenta), following no injection. Solid lines show median of each distribution. Dashed lines show the median of the same distributions but following vehicle administration as is shown in Figure 2A. **(B)** Same as in panel A but for reaction times. **(C-E)** Following no injection waiting times are similarly sensitive to the strength of sensory signal compared to sessions after vehicle administration. (C) Plotted is the waiting time for all trials as a function of SNR following vehicle administration (yellow), and no injection (purple). (D) Same as panel C but only on trials in which the correct choice was made. (E) Same as panel C but only on trials in which the incorrect choice was made. **(F-H)** Reaction time changes similarly following no injection compared to the sessions following vehicle administration. (F) Plotted is the reaction time for all trials as a function of SNR following vehicle administration (yellow), and no injection (purple). (G) Same as panel F but only on trials in which the correct choice was made. (H) Same as panel F but only on trials in which the incorrect choice was made. Error bars show the S.E.M. over sessions and they are usually smaller than the marker. Source data are provided as a Source Data file.

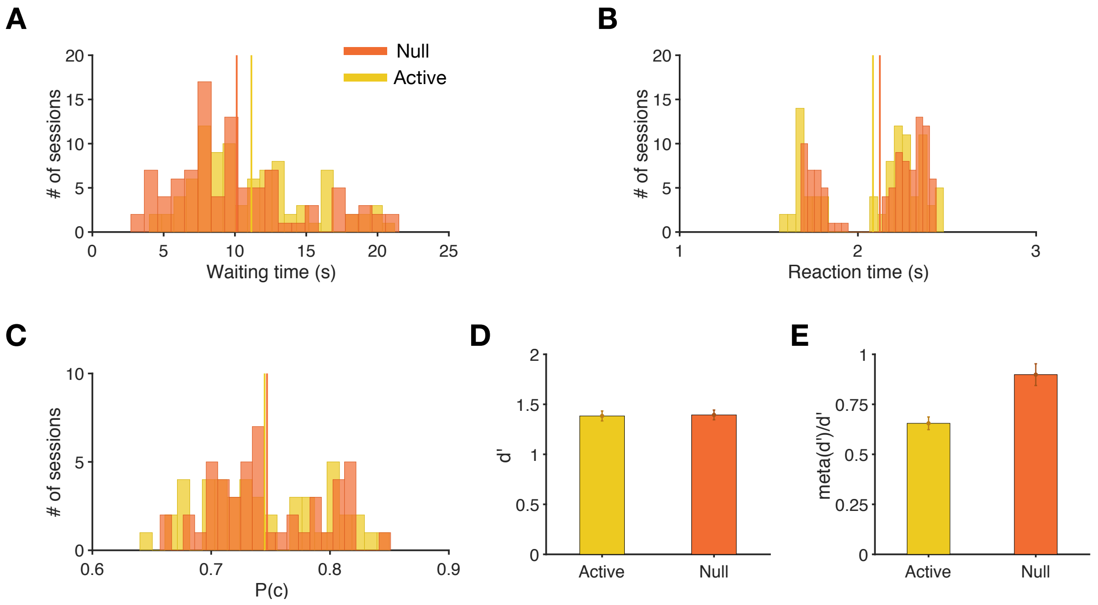

Supplementary Fig. 9. Absence of non-specific effects of virus exposure. **(A)** the type of virus (null vs. active) had no significant main effect on waiting time. Plotted are the distributions of the session-based average waiting time with null (EGFP) and active (DREADDs) virus following vehicle administration in orange and yellow, respectively. The solid lines show the mean of the distributions. **(B)** Similar to panel A but for reaction time. Conventions are similar to panel A. **(C)** Similar to panel A but for the probability of correct response, *P(c)*. Conventions are similar to panel A. **(D)** The type of virus had no significant effect on d'. Plotted is the d' for active (yellow) and null (orange) virus. Error bars show S.E.M. **(E)** Similar to panel D but for meta-d'/d'. Conventions are similar to panel D. Source data are provided as a Source Data file.

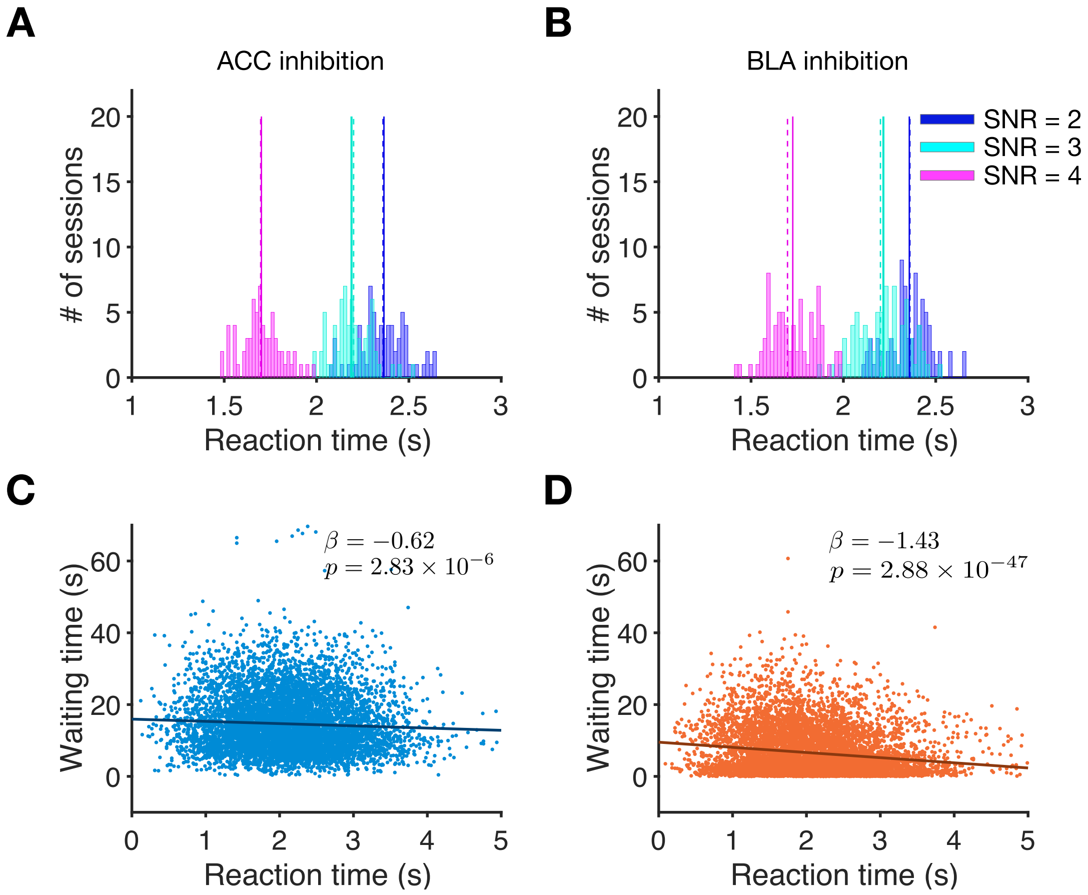

Supplementary Fig. 10. Reaction time is insensitive to inhibition of either ACC or BLA. **(A)** Reaction time does not change following ACC inhibition. Plotted are the distributions of the reaction time for each SNR:2 (blue), 3 (cyan), and 4 (magenta), following CNO administration of ACC. Solid lines show median of each distribution. Dashed lines show the median of the same distributions but following vehicle administration as is shown in Figure 3A. **(B)** Same as in panel A but for sessions following inhibition of BLA, via CNO injection. Solid lines show median of each distribution. Dashed lines show the median of the same distributions but following vehicle administration as is shown in Figure 3A. **(C)** Waiting time before re-initiation of a new trial is negatively correlated with reaction time to make a choice following inhibition of ACC. Waiting time is plotted as a function of the reaction time for all trials and all rats. Each data point is a trial in a session following vehicle administration. **(D)** Same as in panel C but for sessions following inhibition of BLA, via CNO injection. Source data are provided as a Source Data file.

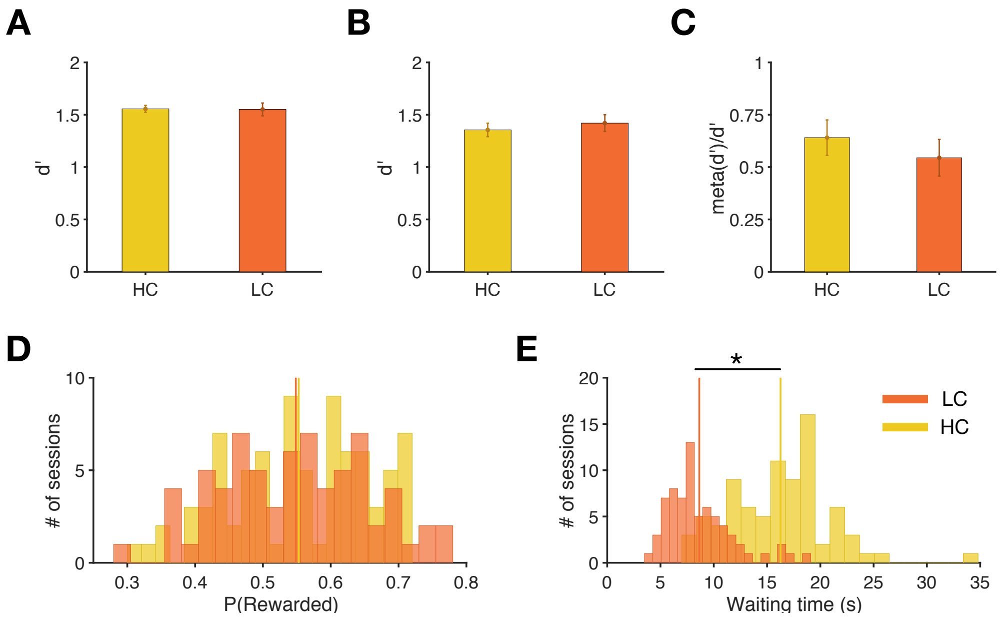

Supplementary Fig. 11. The only difference between HC and LC conditions was the waiting time in the session prior to reversal learning. **(A)** d' for HC and LC conditions were not significantly different for each of the stimuli that was administered after reversal. Plotted is the d' for HC (yellow) and LC (orange) conditions for the contrast-SNR pairs that were chosen for reversal. Error bars show S.E.M. **(B)** d' for HC and LC conditions were not significantly different across all contrast-SNR pairs. Plotted is the d' for HC (yellow) and LC (orange) conditions for all the contrast-SNR pairs. **(C)** Metacognitive efficiency (meta-d'/d') across HC and LC conditions was not significantly different for the specific contrast-SNR pair that was used after reversal. Plotted is the meta-d'/d' for HC (yellow) and LC (orange) conditions for each of the stimuli that was administered after reversal. **(D)** Both HC and LC conditions acquired equal amount of reward in the no-injection control sessions for the specific pair of contrast and SNR values administered after reversal. Plotted are the distribution of the session-based average rewarded trials for HC (yellow) and LC (orange) conditions. **(E)** HC and LC conditions were different in waiting time for the specific contrast-SNR pair that was used after reversal. Plotted are the distribution of the session-based average waiting time for HC (yellow) and LC (orange) conditions. Source data are provided as a Source Data file.

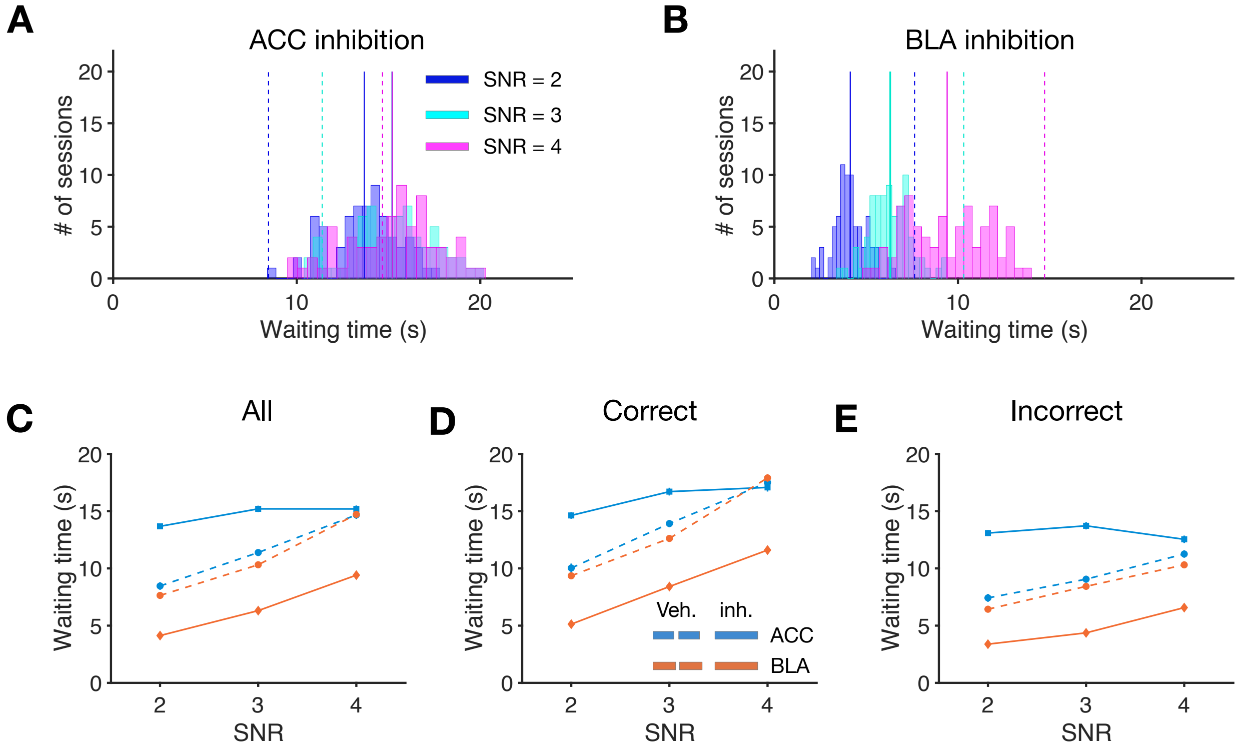

Supplementary Fig. 12. No evidence for difference in waiting time following vehicle administration in either ACC or BLA. **(A)** Waiting time increases following ACC inhibition. Plotted are the distributions of the waiting time separately for each SNR, following ACC inhibition. Solid lines show the median of each distribution. Dashed lines show the median of the same condition but following vehicle administration. **(B)** Waiting time decreases following BLA inhibition. Same as in panel A but for sessions following inhibition of BLA. **(C)** Waiting time for all trials together is similar for the rats with ACC as the targeted brain region following vehicle administration and the rats with BLA as targeted brain region following vehicle administration. Plotted is the waiting time for all trials as a function of SNR following vehicle administration for rats with ACC as the targeted brain region (blue dashed line) and BLA as the targeted brain region (orange dashed line), inhibition of ACC (blue), and inhibition of BLA (orange). **(D-E)** Same as in panel C but only on trials in which a correct (D) or incorrect (E) response was made. Error bars show the S.E.M. over sessions (typically smaller than the symbols). Source data are provided as a Source Data file.

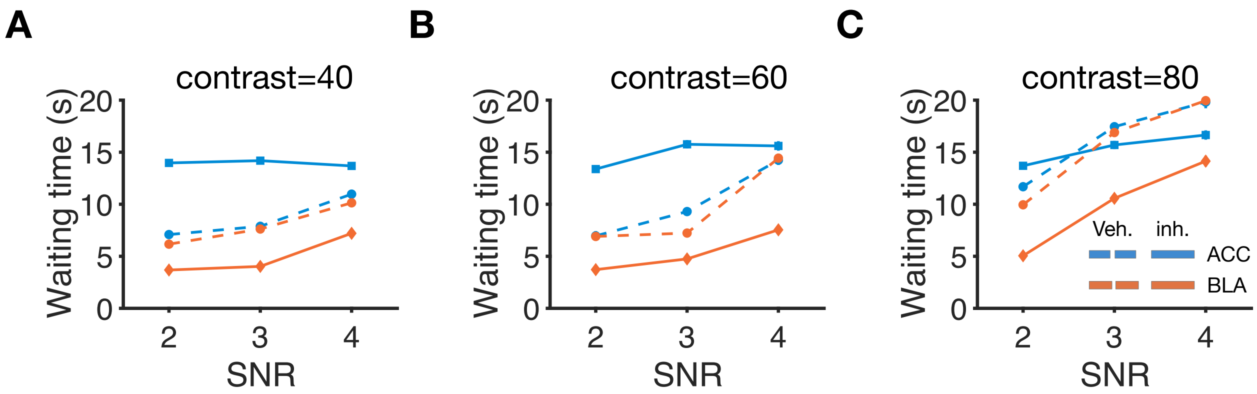

Supplementary Fig. 13. No difference in waiting times for different contrasts levels following vehicle administration in either ACC or BLA. **(A)** Waiting time is similar following vehicle administration in ACC or BLA. Plotted is the waiting time for all trials as a function of SNR and for contrast of 40 following vehicle administration for rats with ACC as the targeted brain region (blue dashed line) and BLA as the targeted brain region (orange dashed line), inhibition of ACC (blue), and inhibition of BLA (orange). **(B-C)** Similar to panel A but for contrast of 60 (B) and 80 (C). Source data are provided as a Source Data file.

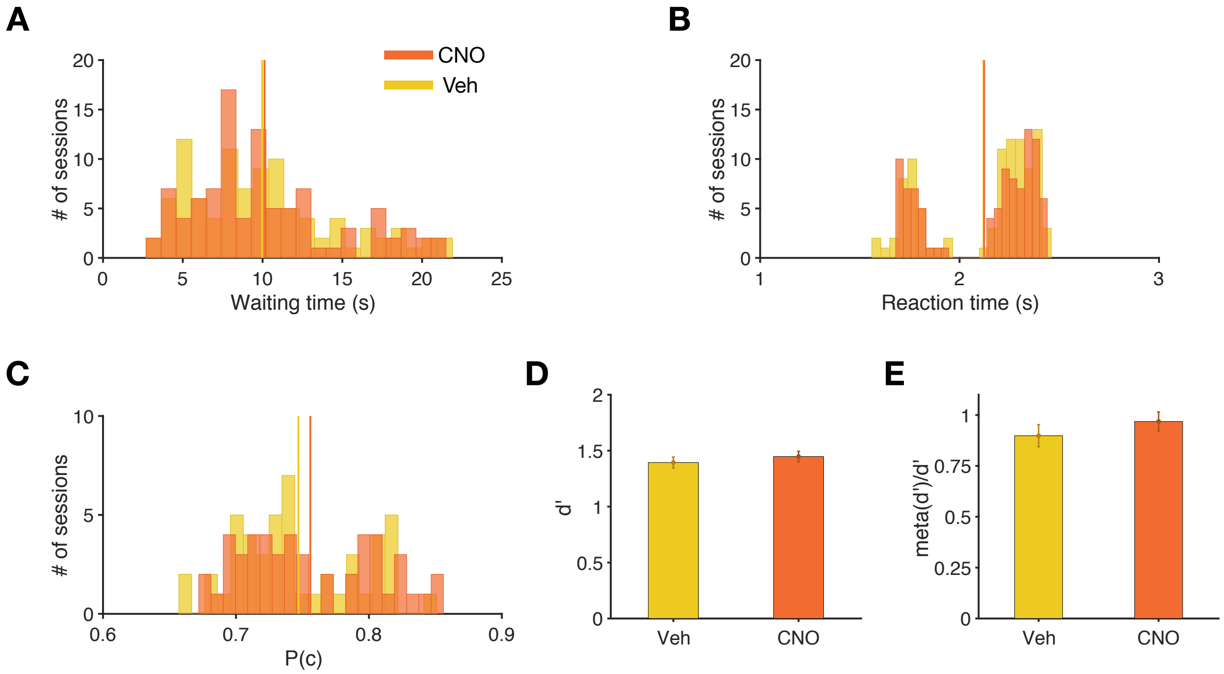

Supplementary Fig. 14. CNO Effects depend on DREADDs. **(A)** CNO had no significant effect on waiting time in the absence of DREADDs. Plotted are the distributions of the session-based average waiting times in EGFP sessions following CNO administration in orange and vehicle administration in yellow. The solid lines show the mean of the distributions. **(B)** CNO had no significant effect on reaction time in the absence of DREADDs. The plot is similar to panel A but for reaction time. Conventions are similar to panel A. **(C)** CNO had no significant effect on performance in the absence of DREADDs The plot is similar to panel A but for the probability of correct response. Conventions are similar to panel A. **(D)** The type of administration in EGFP sessions had no significant effect on d'. Plotted is the d' for vehicle (yellow) and CNO (orange) administration. Error bars show S.E.M. **(E)** Similar to panel D but for meta-d'/d'. Conventions are similar to panel D.
